# ‘Multisectoral socialisation’ in national health governance: An ethnographic study of Singapore’s One Health approach

**DOI:** 10.1371/journal.pgph.0005871

**Published:** 2026-11-03

**Authors:** Matthew Aviso, Youming Ng, James Chan, Panqin Cai, Kelvin Lim, Natasha Howard

**Affiliations:** 1 Saw Swee Hock School of Public Health, National University of Singapore and National University Health System, Singapore Singapore; 2 Communicable Diseases Agency, One Health Office, Singapore Singapore; 3 School of Biological Sciences, College of Science, Nanyang Technological University, Singapore Singapore; 4 London School of Hygiene and Tropical Medicine, Faculty of Public Health and Policy, London, United Kingdom; Institute of Development Studies, UNITED KINGDOM OF GREAT BRITAIN AND NORTHERN IRELAND

## Abstract

The 2025 establishment of Singapore’s Communicable Diseases Agency (CDA) included creation of a One Health Office (OHO), supporting an existing whole-of-government approach involving five agencies spanning human, animal, and environmental health. Singapore’s approach incorporates distinctive features intended to strengthen preparedness, prevention, and mitigation during non-emergency periods. We examined how this approach developed and implemented multisectoral governance of national One Health priorities. This nine-month ethnographic study (2024–2025) involved observations of three inter-agency One Health meetings and eight partner agency engagements, semi-structured interviews with 26 public servants across five partner agencies, and review of eight internal government documents. Data were analysed thematically using abductive coding. We found that Singapore’s One Health governance was supported by participant-driven socialisation through ‘semi-informal’ inter-agency relations among public servants. These were reinforced by a rotational governance structure, Singapore’s small size, regular bilateral and multilateral communication, and OHO-led engagement. These interactions enabled workable inter-agency arrangements while progressively strengthening multisectoral governance. Importantly, socialisation processes appeared structurally supported rather than emerging incidentally from formal governance arrangements, challenging conventional assumptions about the relationship between formal structures and informal networks. Singapore’s One Health approach demonstrates how multisectoral governance can be deliberately strengthened through interpersonal socialisation. Formal institutional arrangements, including those established through CDA, can cultivate ‘semi-informal’ networks that foster trust, shared ownership, and consensus across government agencies. For national public health agencies elsewhere, embedding opportunities for sustained socialisation within governance structures may strengthen intersectoral collaboration and collective ownership of One Health priorities. The Singapore experience suggests that informal social processes need not be incidental outcomes of formal governance but can be intentionally enabled through institutional design, with implications for the development and assessment of One Health governance internationally.

## Introduction

Multisectoral governance refers to coordination and collaboration among several sectors (e.g., health, agriculture, environment, urban planning) to address complex challenges that span disciplinary and organisational boundaries, emphasising collective decision-making, shared responsibility, and integrated responses to achieve common goals [1,2]. The city-state of Singapore has intentionally developed multisectoral partnerships for health governance, with Singapore’s successful COVID-19 response demonstrating the value of multisectoral and ‘whole-of-government’ (WOG) approaches. The 2023 COVID-19 White Paper proposed strengthening these approaches for pandemic planning and preparedness, given the increasing frequency of infectious disease outbreaks and antimicrobial resistance [3]. Spurred by noted gaps in inter-agency partnerships in the national COVID-19 pandemic response, Singapore established an interim Communicable Diseases Agency (iCDA) in 2024—a pre-operational model to facilitate transition into the Communicable Diseases Agency (CDA) [4]. CDA became operational in April 2025 as a national public health agency (NPHA) that functions as statutory board under the Ministry of Health (MOH) and consolidates public health functions previously spread across the Ministry of Health (MOH), National Centre for Infectious Diseases (NCID), and Health Promotion Board (HPB) [4]. Since establishment, CDA has functioned as the national infectious diseases authority, with MOH overseeing Singapore’s health system, NCID strengthening clinical preparedness and response services, and HPB focusing on disease prevention. Some shifts in functions include the Antimicrobial Resistance Coordination Office moving from NCID to CDA [5].

CDA functions both as part of and autonomously from MOH, serving as the frontline government agency to prevent, prepare for, detect, quickly respond to, and enable research in infectious diseases in Singapore. This includes leading and coordinating public health preparedness; safeguarding national interests such as vaccine and therapeutics development; strengthening surveillance capabilities through new modalities such as wastewater testing, data analytics, and artificial intelligence; and conducting contact tracing and border control measures [6]. Singapore’s MOH leadership indicated that CDA will provide policy and scientific recommendations [4] as part of ongoing multisectoral approaches to addressing current and emerging public health threats. While CDA is still in its infancy, it is helpful to examine pre-existing multisectoral health governance efforts in the country to inform governance models for CDA and other NPHAs. This study thus focuses on the new One Health Office (OHO), a CDA division, to explore the development of multisectoral governance approaches in Singapore.

‘One Health’ as a collaborative and integrative approach to the health of humans, animals, and ecosystems was central to the Joint Strategic Framework developed by the World Health Organization (WHO), World Organization for Animal Health (WOAH), and Food and Agriculture Organization (FAO), in collaboration with World Bank, UN System Influenza Coordination (UNSIC) and UNICEF in 2008 [7,8]. WHO, WOAH, FAO became the Tripartite in 2010, with United Nations Environment Programme (UNEP) joining in 2022 to make it the ‘Quadripartite.’ Multisectoral coordination is a foundational principle of the One Health approach, emphasising inclusivity, accountability, and coordination among diverse human and animal health stakeholders, to improve decision-making and resource usage in addressing human, animal, and ecologically related disease threats [8]. From avian influenza to Ebola and, more recently, mpox, linkages between humans, other animals, and the environment require multisectoral action to prevent and mitigate emergencies [9–12].

Recognizing this need for multisectoral collaboration, Singapore established a formal inter-agency One Health framework in 2012, including MOH (now through CDA), the National Environment Agency (NEA) to mitigate impacts of vector-borne diseases such as dengue fever, Singapore Food Agency (SFA) for food safety, National Parks Board (NParks) for diseases that may affect both animals and humans, and Singapore’s National Water Agency (PUB) for water safety. This initiative seeks to address emergent global health challenges in an era of climate and ecosystem changes that have displaced multiple animal species, fostering the rapid development of microbial beings that, in the worst case, become pandemic-level threats [13]. Significant progress included developing joint response protocols for priority diseases, strengthening workforce capacity, and establishing risk communication and surveillance workflows [14,15].

The COVID-19 pandemic prompted Singapore to strengthen One Health measures and re-envision this approach, leading to OHO’s establishment in early 2024 as part of the then-iCDA. Under CDA, OHO aims to bolster intersectoral governance, policy, and advocacy development. Since its inception, OHO has coordinated stakeholders from the five national lead agencies to develop strategic direction and measurable actions for the One Health space. Comprising permanent and seconded staff from the human, animal, and environmental health government sectors, OHO is designed to facilitate smooth coordination with non-OHO One Health stakeholders in Singapore. This cross-sectoral staffing itself embodies One Health principles, as personnel from different sectors bring diverse perspectives to the coordinating body. Towards that end, OHO developed a One Health masterplan to safeguard national public health, representing the culmination of collaborative efforts across Singapore’s health, environmental, and animal sectors. Published in late 2025, the masterplan was developed based on multiple consultation and consensus-building inter-agency meetings attended by human and animal disease control professionals, ecologists, and those working in related disciplines, and intended as a living document that could incorporate stakeholder feedback over time [16]. The masterplan operationalises One Health outcomes through network and leadership engagements, sector development via capacity building, and multisectoral research collaborations, demonstrating how One Health is defined through practice rather than only through formal definition. Via these activities, CDA intends to establish itself through OHO as a coordinating body for multisectoral One Health collaborations across partner agencies and external stakeholders.

We aimed to analyse how the OHO and Singapore’s One Health approach develop and implement multisectoral governance of national One Health priorities. Objectives were to: (i) describe the structural processes through which the One Health approach fosters multisectoral governance efforts among different agencies; (ii) explore how inter-agency One Health meetings demonstrate multisectoral governance processes; and (iii) identify key lessons that can inform national and global multisectoral governance of NPHAs.

## Methodology

### Study design

We adopted an ethnographic study design [17,18], drawing from descriptive and exploratory data gathered from semi-structured interviews [19], participant observations, and document review. Ethnography is a fieldwork methodology that attempts to collect “thick” descriptive data in the form of observational fieldnotes to describe the “insider” perspective of a social or cultural group. It is particularly suited for collecting detailed qualitative data related to social interactions and processes and involves reflexively negotiating the impact of one’s “outsider” presence on the proceedings of the observations [20]. Rather than coming from a position of objectivity, ethnography acknowledges that all qualitative data are inherently subjective and cannot be elicited or extracted in a controlled setting as laboratory data can. Therefore, in contrast to a methodologically similar study conducted on health policy spaces in Singapore that considers itself as engaging in “non-participant observation” [21], we consider ourselves participant observers whose presence cannot be divorced from the field within which we make our observations. Our research question was: “How does Singapore’s One Health approach help develop or strengthen multisectoral governance?”

### Data collection

#### Participant observation.

One investigator (MA) participated as an observer in a series of OHO-coordinated inter-agency workshops and meetings to describe the interactions and activities occurring in these spaces. He prepared ethnographic fieldnotes of proceedings, including observations and conversations among attendees. Semi-structured observations (e.g., of verbal intonation and body language; affirmative or disconfirming assertions; acts of disengagement, challenge, or submission) and ethnographic conversations were noted through traditional fieldnote-taking. Ethnographic conversations are unstructured and iterative by nature and thus did not follow a pre-prepared question guide, instead documenting and clarifying important or interesting points that may not be obtained through observations or formal semi-structured interviews. These conversations arose spontaneously during meetings, were not audio-recorded, and were used to inform interpretations of observations and revise lines of questioning in the semi-structured interview guide.

From 1 September 2024–30 June 2025, MA (as participant-observer) attended two One Health scoping workshops for masterplan development and one meeting for action plan drafting, with eight site visits to representative agency offices where observational notes were taken alongside in-person interview notes. OHO and partner agencies provided permission to attend meetings or visit offices for observation and conversation, with MA’s meeting attendance limited to multisectoral scoping meetings. Each OHO-coordinated event MA attended lasted three to seven hours.

#### Key informant interviews.

We developed a question guide based on One Health governance literature [22,23], initial observations, and review of internal documents provided by CDA. It included topics on national One Health governance approach, institutional and personal relationship with CDA and OHO, limitations and possibilities of inter-agency One Health work, shared experiences of inter-agency gatherings (in which MA clarified participant thoughts on key meeting moments to also corroborate observation fieldnotes) and experiences of meetings MA did not attend. These last constituted a form of oral history often employed in ethnographic approaches [24].

We used purposive maximum variation sampling [25,26] as the most feasible way to sample across the small pool of 65 candidate public servants, based on a list of attendees in inter-agency One Health gatherings since late 2024. Representation from across the five agencies in these meetings are roughly equal in terms of attendee count. Eligibility included public servants who: (i) had worked in Singapore public service for a cumulative three years prior to interview, and (ii) attended at least one inter-agency One Health gathering as participant or facilitator between 2024 and 2025. Variation was prioritised along three axes: (i) hierarchical level within the One Health governance structure (executive/junior staff, director-level, group director/senior management), enabling comparison of how socialisation processes differed across power gradients; (ii) agency affiliation across the five partner agencies (i.e., CDA, NEA, NParks, PUB, SFA), capturing sectoral perspectives from human health, animal/wildlife health, environmental health, food safety, water safety; and (iii) length of engagement with One Health activities (new participants versus those involved since the 2012 framework), allowing temporal comparison of socialisation depth. This sampling strategy ensured we captured perspectives from those with institutional memory of the pre-OHO era and newcomers experiencing the formalised structure. Public servants were initially approached in-person at One Health meetings. Due to the dynamic nature of public service, scheduling constraints limited the number of initial responses. We then employed snowballing, whereby participants were asked to recommend other participants who we contacted via email or in-person during succeeding One Health gatherings. This facilitated the identification of candidates and inclusion of individuals with specialised knowledge or unique perspectives that may otherwise have been overlooked.

We provided potential interviewees with the study information sheet and opportunities to ask questions before recording written or verbal (as participant’s preferred) consent before interviews. MA contacted 31 potential participants, ultimately conducting 21 interviews (18 individual and 3 group) with 26 key informants between 6 March and 31 May 2025, at places of participants’ choosing (typically their offices) or remotely using Zoom (Zoom Video Communications Inc, San Jose, California) or Microsoft Teams software. Interviews were audio-recorded through Zoom or a separate audio-recording device, with automatic transcription enabled. One participant was interviewed through writing per their request, while five did not wish to be recorded but allowed notetaking. Interviews lasted 45 minutes on average, with MA reviewing and correcting transcripts after each interview.

#### Document review.

We reviewed 8 internal documents provided by OHO, to provide contextual understanding of interviews and observations. Documents included slide decks presented in meetings the team was not invited to attend, summaries of proceedings, participant lists, updated terms of reference, and drafts of One Health planning documents. No classified or restricted information is reproduced here.

### Analysis

Analysis was conducted simultaneously with data collection and prioritised anonymisation of study participants, given the small pool and easy identifiability. MA analysed data in NVivo 11 software (Lumivero LLC) employing Braun and Clarke’s thematic analysis [27] with an abductive approach described by Thompson [28,29]. This integrated deductive coding based on the question guide while leaving analysis open to new insights. It included in-depth (re)reading of transcripts, initial open coding, refinement into broad themes, recombination, and interpretation/contextualisation. Interpretation and contextualisation were aided by review of internal OHO documents. An abductive approach was most appropriate to address our initial set of questions exploring specific structural features of the One Health space while enabling new ideas related to socialisation processes that challenged our initial conceptual assumptions. Acknowledging that abductive analysis in ethnography entails that initial assumptions and questions change and develop through the course of fieldwork, later interviews thus included questions and discussions touching on tentative thematic analysis, constituting a form of participant checking. Additionally, anonymised coded data were shared with CDA co-authors for contextualisation to aid interpretation, though final interpretive decisions rested with MA and NH to preserve critical distance. This collaborative-yet-independent approach balanced insider contextual knowledge with outsider critical perspective as a deliberate methodological choice.

### Reflexivity

The participant-observer (MA), a male Filipino early-career scholar trained in the anthropology of bureaucracy, approaching One Health from a governance rather than public health orientation. This outsider orientation, along with MA’s relative youth and limited public health exposure, may have filtered event attunement and analytical attention, a limitation we addressed through collaborative contextualisation processes described above. We mitigated this through collaborative contextualisation amongst authors.

CDA co-authors (YN, PC, JC, KL; three male, one female, all Singaporean, from environmental, animal, and human health backgrounds at mid-to-senior career levels) enabled access by identifying meetings, introducing MA as partner researcher, and sponsoring entry into closed governmental spaces. This sponsorship eased access but fostered initial wariness about reputational risks among participants. Rapport grew through repeated interactions, referrals, and prior positive experiences with the senior investigator (NH, female Canadian scholar-practitioner with health systems and infectious disease expertise) mitigating perceived power asymmetries.

CDA co-authors supplied attendee lists for sampling but took no part in recruitment decisions and no identifiable data were shared with them. Their analytical role was limited to contextualising anonymised excerpts, regarding institutional processes and historical background that outsiders might misinterpret. Final interpretation rested with MA and NH, to preserve critical distance.

NH, as senior researcher, provided public health contextualisation, methodological and operational supervision, and interpretation. We encountered productive tensions between MA’s governance-oriented interpretation and CDA co-authors’ institutional perspectives, which were resolved through discussion while maintaining analytical autonomy. This collaborative yet independent approach balanced insider contextual knowledge with outsider critical perspective.

### Ethics

The National University of Singapore Institutional Review Board provided ethics approval (reference NUS-IRB-2025–41). Several measures protected participant confidentiality given the small, easily identifiable pool and CDA co-author involvement, representing best practices for studying small, elite government populations where standard anonymisation techniques may be insufficient. First, while CDA co-authors provided lists of meeting attendees, they were explicitly not informed which individuals were contacted, who agreed to participate, or who declined, creating space between institutional gatekeepers and research participants. Second, interview transcripts, fieldnotes, and coded data were stored on password-protected systems accessible only to participant-observer and senior researcher. Third, anonymised excerpts shared with CDA co-authors for contextualisation were stripped of identifying details beyond agency affiliation (and where even agency affiliation might be identifying in very small agencies, were further aggregated or paraphrased). Fourth, we avoid reporting specific numbers of participants per agency, specific roles that might identify individuals, and anecdotes referencing particular responses or events that could narrow identification. Fifth, participants were explicitly informed of these protections during consent processes and could choose to be interviewed in writing, decline recording, or stop interviews at any point.

## Findings

### Participant characteristics and themes

We interviewed 26 participants, 18 individually and 8 in three group interviews, with proportional representation across the One Health structure. Relatively equal numbers of interviewees across agencies were engaged, with specific numbers not provided to protect the confidentiality of the small number of people from each agency. Several junior or staff-level participants were previously engaged in committees drafting the One Health masterplan and action plan. We also limited details on agency and other identifying details to help ensure anonymity in this small community. Five potential interviewees declined participation, instead connecting us with other One Health stakeholders to interview.

We generated four abductive themes: (i) distributing equal ownership through rotating chairmanship; (ii) centring communicable human diseases; (iii) fostering ‘semi-informal’ relations; and (iv) deepening connections through coordinated bilateral engagement. Findings below reflect participants’ perceptions of Singapore’s One Health approach.

### Distributing equal ownership through rotating chairmanship

Fig 1 shows the different One Health structure levels after CDA formation, accurate during the period of data collection, with all participant insights and opinions based on experiences with this structure. Staff participants at director-level and below were asked by OHO for inputs on the One Health masterplan and action plan, and these same members were organized to develop action items. The secretariat served as focal point through which programmes and communications were coordinated from OHO to relevant stakeholders in their home agencies. OHO functioned by design as secretariat for the CDA. The One Health Working Group (OHWG) consisted of members from senior management (i.e., Group Director-equivalent) who developed and coordinated programmes and activities based on staff-level input. The One Health Coordinating Committee (OHCC) oversaw the One Health space, consisting of members of senior management (i.e., Director General-equivalent) that review and endorse programmes and activities proposed by the OHWG and make final decisions based on consensus.

**Fig 1:**
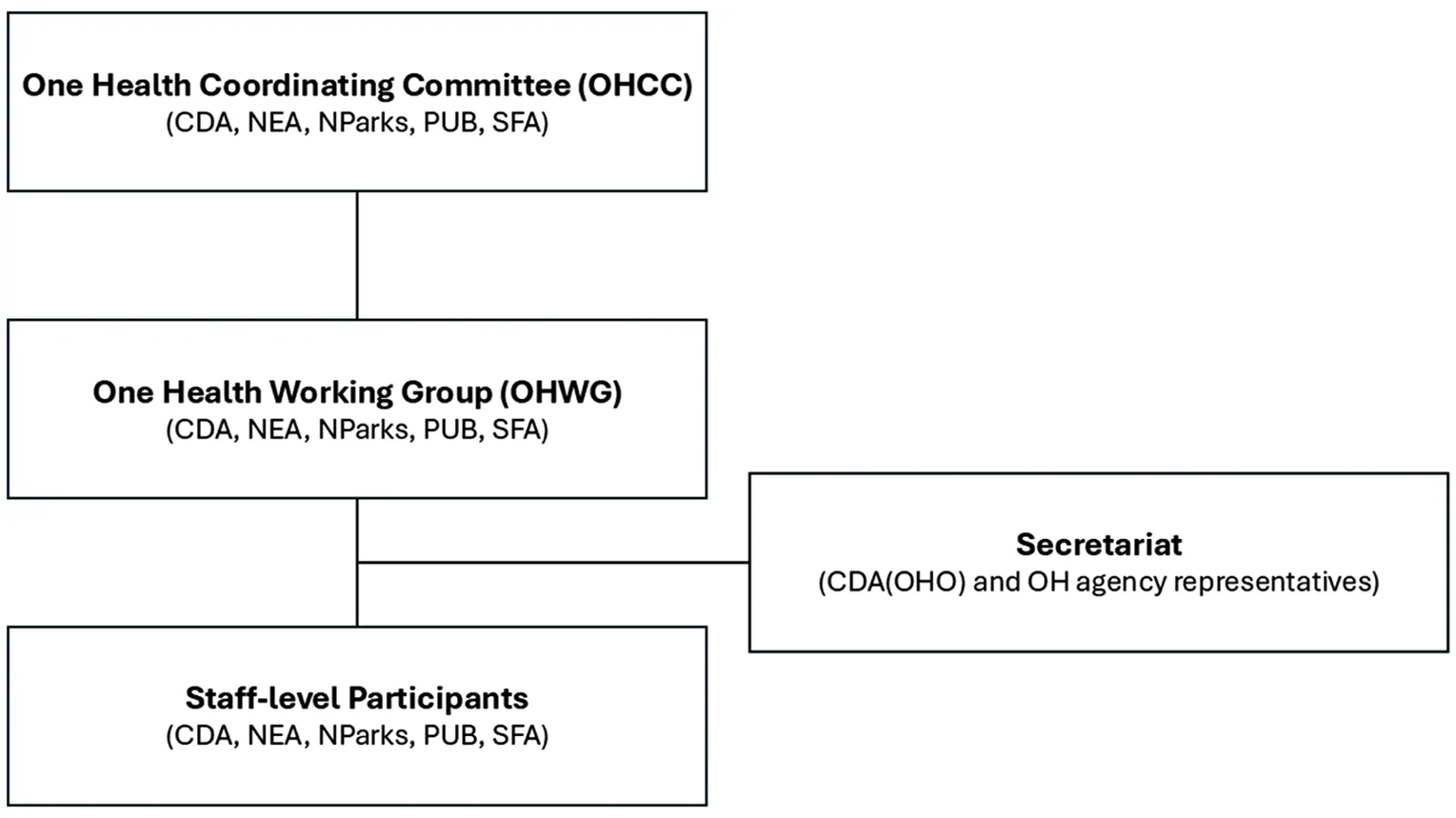
Singapore One Health structure (as of August 2025).

Rotational chairing was a structural feature of the Singapore One Health approach since 2012. This meant that different agencies took turns annually to chair meetings and set agendas. Even before OHO formation, rotating chairmanship was considered a “signal of neutrality.” This was deemed particularly important for non-crisis work, as opposed to just letting one agency such as MOH (or now, CDA) take the lead, as argued by an interviewee:

Having a lead is great for crisis when there are a lot of disagreement because somebody will decide and everybody falls in line. But at the same time if you only have one agency, the other agency might say, “Hey, I’m not responsible.” So, it’s the other agency’s responsibility. Would they scale down some of their involvement? But because it’s rotating, everybody has a responsibility.

This culture of rotation across levels and agencies, however, is not new in Singapore public service. For junior-level officers, rotations allow them to be exposed to different areas of work within their home agency or others. For instance, within agencies, the secretariat rotates among junior staff, though specifics may differ by agency. As a participant described:

People movement is important, and I’m not sure whether other countries do that [for junior staff], where they have cross posting of people […] For us, it’s for grooming and development but it’s also for understanding the other agencies as well.

Even senior management are rotated frequently in Singapore public service. Such a rotational system was affirmed by many participants as a strength in national governance structures, as transferring diverse technical experts around different portfolios in different agencies helped the cross-fertilisation of ideas—a feature that is especially important for One Health, which is collaborative and complex by design. One senior official advocated for this model particularly strongly:

Something that I’ve noticed that is quite unique in Singapore…and something I have also recommended and pushed quite a lot internationally is how common[ly] staff and people get rotated. Yeah, I think that it’s very common [that] medical doctors are inside in MOH. Then the veterinarians are in other agencies […but now there’s] a vet [seconded] to MOH to do One Health. And there are many of this going on, especially when it comes to leadership. Our CEO will move. So it can be very tiring because you keep changing CEO. But as I see the system, as I grow, as I grow older, I see the power of this movement […These CEOs] also bring our agenda to the next ministry [they handle]. So there is a lot of mutual understanding between agencies because of this movement of people. If you stay put in one area for too long—twenty, thirty years—you become too guarded.

Still, some acknowledged that rotating chairmanship could have unintended consequences for perceived responsibility in One Health, with one participant noting:

It’s always circled back to the discussion that it’s an equal ownership of One Health and therefore we do the rotating thing. But if everyone owns it equally, sometimes nobody owns it because, everyone’s kind of like…. it’s all our jobs. You know what I mean? Like if you don’t put someone and say that they’re the ones in charge, then it’s hard to get together a concerted effort.

OHO formation sought to resolve the issue of dispersed rotational efforts by centralising the bulk of coordination, administration, and documentation work while still ensuring each agency took turns annually to chair activities and table One Health items. The OHO functions as central secretariat, with a senior official describing its presence as making sure “there’s some longevity and drive to maybe some of the major initiatives that the agencies may want to pursue,” although it will not necessarily drive technical leadership and expertise. However, OHO staff themselves were also subject to rotations to other functions within or outside CDA under the Singapore public service system. Nonetheless, as OHO was newly formed, staff numbers and service lengths were malleable to provide flexibility as the office established itself.

### Centring communicable human diseases

OHO was young and its secretariat staff strove to demonstrate in meetings that they were coordinators and not just there to provide the CDA (i.e., human infectious diseases-centred) perspective. One participant observed:

At least from an optics point of view, One Health Office makes a point to distinguish themselves from [the rest of the CDA] in the sense that when they run a meeting that’s just the Secretariat, they don’t offer the opinions as a One Health body.

Nonetheless, many participants indicated that the CDA should house OHO as it is only appropriate that a human health-centred entity should be the structural centre of Singapore’s One Health space. As one interviewee noted:

I personally think it’s correct to be under MOH [via its statutory board CDA], all right… As an urban city, it is really human density that is high. So, if there’s any disease transmission, it will be human. So, I think from the, especially when it comes to the Singapore perspective, [One Health being centralised] under MOH is correct.

However, some also expressed concerns that focusing on infectious diseases in humans and pandemic preparedness could limit discussion of other important One Health issues.

Just one possibility, because it’s under the Communicable Diseases Agency […] there may be other One Health threats that are not communicable. So […], if it becomes a need, [we need to discuss] whether that would be something that CDA is comfortable with.

For instance, one participant wanted to centre chemical pollution’s impact on human health in future One Health meetings:

Environmental pollutants or contaminants that are starting to affect human health as well because of our exposure to them […] These are also one health areas. But I don’t think that we have been talking about these enough. I think we’ve only been talking about viruses and bacteria causing diseases […] I feel that, you know, for Singapore, maybe we also want to have a conversation about… do we also start to look at these other areas besides the conventional bacteria and viruses?

Another interviewee was optimistic that the institutional structures in place and the relationships these fostered would make discussion regarding such grey areas within national One Health priorities fruitful:

I think with CDA coming in, we have very much scope to look into more of the infectious disease space. But given that we have about 10 years of working together, even though it is not infectious disease, [even if] it could be a chemical hazard that might involve a couple of the agencies, I think that that familiarity and that way of talking and working does come in even for things that might now fall outside the [human infection-centred] One Health.

The concern that CDA’s human infectious disease focus might marginalise animal and environmental health priorities was legitimate and voiced by some participants. However, several countervailing mechanisms were noted. First, rotational chairing ensured each agency had regular opportunities to set agendas and table sector-specific concerns, providing structural protection against permanent marginalisation of non-human-health perspectives. Second, the socialisation processes, built over a decade of collaborative work, appeared to have fostered sufficient trust and mutual understanding that participants expressed confidence in their ability to negotiate priorities even when human health leads. Third, OHO’s multi-sectoral staffing (personnel seconded from environmental and animal health backgrounds) provides internal advocacy for non-human-health perspectives within the coordinating body itself.

### Fostering semi-informal relations

Many participants described the abovementioned “familiarity” and relative ease with which they could connect with others as characteristic of a “semi-informal” relationship—described as being able to maintain professional boundaries while still being friendly and accessible to each other. Most participants suggested that such semi-informal relationships were possible because the One Health space was formed by putting together a small group of participants across the agencies with mutual interest in cross-disciplinary public health work. A participant claimed that the “soft” quality of this Whole-of-Government initiative enabled compromises and agreements to be easily reached even across multiple sectors:

It actually allows us to speak our views, maybe sometimes a bit more freely than if it’s a real, you know, formal meeting where, now whatever I say represents [my agency] and so sometimes, we are able to just sound out some of the ground issues quite openly. Yeah, I would say, my sensing is that even at the higher level, up to the Director-General level, they are obviously more formal than us when they come together for our meeting, but they are not as formal as some of the meetings that I’ve seen. So, they are reasonably formal, but they also maintain quite open conversations. They can give differing views, but they always seek a consensus in the end.

This consensus-seeking process enabled the formation of semi-informal relations that some participants referred to as a process of ‘socialisation.’ Right after an inter-agency gathering that included unresolved debates about task distribution, an attendee told MA that since every participant was busy, it was difficult to prioritise tasks and make sure there was a sense of ownership for all parties. The participant said that this sense of ownership would eventually be reached, it just required what they called “socialisation.” When MA asked a different attendee what it means to ‘socialise’ a sense of ownership, they said that it was about “slowly bringing people into consensus.” For this person, it was natural for people to express disagreements when the “buy-in” was not yet clear. The process of socialisation involved a series of ongoing discussions and related conversations that led from disagreement to a compromise and eventually also a renewed sense of ownership of the One Health space that compelled these participants to proactively engage in collaborative work.

So when you socialise, we got to think three steps ahead… because we may not have socialized the idea initially. We went straight to step four when in their world, they are like, “What’s this? Who’s this? What am I supposed to do? How come it’s me?” Yeah, those four questions, right? We need to go four steps before to make it very friendly from their point of view to neutral, almost not uncomfortable starting point. Slowly bring them to the step four. Not jump straight to [step four].

This socialisation process and development of semi-informal relationships thus co-construct each other. Through its years of socialisation, the One Health space has moved beyond formal and professional bilateral partnerships to a uniquely warm collegiality observable in the five-agency gatherings. Before workshops and meetings officially began, there was active chatter, laughter, and discussion among participants across different agencies. Such informal discussion sometimes cut across different structural levels, with senior officials mingling with junior staff. The ensuing semi-informal relations made consensus easier to achieve. According to participants, this warmth of relations took years to develop but arose primarily due to formation of the One Health space, as one public servant described:

In the early days, each agency will kind of deal with their own issues and if there is anything that requires any other agency’s help, it’s usually quite formal. It’s through email and then followed by the meeting. And it was very specific issues of concern. And once that is done, it’s like, you know, there’s no further engagement, unless you have further engagement required with that agency. So, when we first started […] it was more a few directors coming together to talk to each other first. Like over a cup of coffee with each agency, having that agreement to talk about whether you will be keen if you want to start off this thing.

This culture of informally catching up on potential collaborations over coffee or tea during breaks eventually became a mainstay in the One Health work culture, no longer just among directors but also at all staff levels. As an interviewee put it, this culture enabled people to “convene quickly” in case “an urgent situation arises.” A senior official shared the same sentiment:

I think most important when something happens, you need to know who’s that person you can call up. Do you have that person on your “speed” dial [laughs]? On your hand phone? And do you reach out? So I think that it’s a softer side of the thing of the house. It’s not like concrete.

For all participants, the development of such “softer” relations amid otherwise formal professional relations was the value of putting together these five agencies in multi-agency gatherings:

The big group meetings also have one benefit in that you actually learn to put the faces to all the names and it really helps that it facilitates […] rather than I just send an email cold to someone, right? And they don’t know who I am […] I can now at least say like, “Oh, we met at, you know, that One Health meeting last month and, you know, I got this issue and, you know, can we have a chat about this, you know, or something?” So, I think that that is enormously helpful.

These semi-informal relations allowed for smoother coordination when it came to health emergencies such as the Covid-19 pandemic or recent oil spill, with one participant calling the government response to the former a “smooth running of gears” due to the capacity and relationships built in the One Health space. One senior official affirmed this, saying:

There is actually high level for support that we give each other. Me to my counterpart and vice versa. You know, the counterpart when I asked them for help, they are actually quite prepared to support as well […] So I think that’s the first point, that we do have a structure in place to facilitate this working across agencies. And then the other thing, I think, is very useful is that we do know, I mean, personally, I’ll say that I do know most of my counterparts. So not only is there official structure, there’s also the personal networks that has formed over the years because we work with each other on various things like I mentioned [...]. So, I actually got to know [people] better or got to know first through One Health and then after I got to know [them] better as we were on some other crisis that we faced along the way.

Participants described how relationships built through One Health socialisation enabled rapid informal coordination alongside formal structures during COVID-19 and an oil spill incident. The informal networks activated during crises complemented rather than conflicted with formal emergency protocols, suggesting that socialisation during peacetime created relational infrastructure that enhanced, but did not replace, formal emergency governance. During non-emergency periods, rotational chairing distributed leadership and agenda-setting across all five agencies, fostering equal ownership but potentially diffusing responsibility. However, participants clarified that during public health emergencies, Singapore has clearly defined, though confidential, command protocols establishing chain of command, typically with MOH/CDA in lead coordinating roles given the human health primacy in most emergencies. The tension lies not in confusion during crises but in maintaining urgency and preparedness investment during “peacetime” when rotating leadership may reduce any single agency’s sense of ultimate accountability. This is the gap OHO now fills, providing continuous coordination infrastructure while agencies rotate through leadership roles.

### Deepening connections through coordinated bilateral engagements

OHO, itself consisting of participants in the One Health space, attempts to build on and develop these semi-informal relations through increased bilateral engagement with all participants to discuss points of internal debate in more detail, providing a personal touch in the One Health space. As one participant described:

On top of that multi-group meeting, [OHO has] been doing bilateral engagement as well and identifying our needs and see how we can put our agenda in there. So that, I think, is a very fantastic strategy. I will say it is very sincere. [They] came to us and asked us about what our needs are […]. Having a mandate, putting an office where people do it full time, I think really has enabled…this kind of connection.

Bilateral meetings facilitated efficiency and streamlined multilateral discussions by clarifying and focusing agenda items. As a senior official said:

The bilateral meeting helped because when you go to a meeting with four [other] parties and some comments would be quite specific […If we did not have bilateral meetings with the OHO], I think we’ll probably be sucked into a little bit more minor details.

With trust built through inter-agency meetings and regular semi-informal interactions, many participants expressed how easy it had been to coordinate bilateral discussions simply and efficiently with OHO. Rather than calling them meetings, most preferred to call them “catch-ups” or “chats.” One participant explained:

At the smaller group level, I’m quite glad that I guess we are a bit less formal and we could reach out through [virtual call] and whatever. And that also explains why we have quite a number of ad-hoc meetings sometimes. But some of the meetings were just very focused. It’s very short. We just iron out any issues. And then I will say I appreciate everyone reaching out […] to the different stakeholders and then just asking us, “What are your issues? Do you want to talk it over a 15-minute call?” And we manage to iron quite a number of issues through that. And it’s, it’s quite efficient as well.

Over time, the “friendliness factor”—as one interviewee described it—of OHO increased and it became easier to raise such feedback in other bilateral discussions. Participants appreciated these bilateral engagements. However, some also noted that while such “friendly” approaches were helpful in building connections and a sense of ownership they risked being overly “personality-driven,” as another participant put it. This participant mentioned a degree of “reliance on personal factors” in such outreach, as “some work is dependent on the ground-up efforts of vested individuals, which may not be sustainable in the longer-term or if there are changes in personnel.” This suggested that relational and formal governance mechanisms must be balanced to ensure sustainability.

## Discussion

### Socialising multisectoral governance

This study found that developing multisectoral One Health governance in Singapore was predicated on socialising ‘semi-informal’ cross-institutional relationships and ownership. Multisectoral One Health governance was initiated through formal structures but enacted through social networks. The social was thus central to governance, which was “grounded in interaction” [30]. Given ethnography’s suitability for studying interactions, our initial conceptualisation focused on interactions between structural (e.g., regulations, protocols, bureaucratic processes) [22] and social dimensions (e.g., meeting behaviours, interpersonal communication within and across networks) [23] of One Health governance. However, inductive findings revealed how crucial informal social relations are in Singapore’s One Health space, and how organisational policies - including rotational chairing, compact institutional landscape, and regular multi-agency gatherings - appear configured to foster these relations.

Rotational chairing fostered these social networks, giving each of the five partner agencies opportunities to chair meetings and set agendas. Based on Singapore’s public service culture of rotating personnel across agencies [31,32], this system encouraged shared responsibility, leadership experience, and adaptation to differing work cultures. While beneficial, dispersion of responsibility raised some participant concerns that no single agency holds clear leadership responsibility. Clear protocols (kept confidential) are in place for chains of command during periods of public health emergency, but developing a sense of urgency for leadership can be more challenging during relatively quiet periods. OHO’s creation as secretariat helps address this tension, by functioning as the main coordinating body across rotating chairs. Centralising One Health governance under CDA, a human infectious disease-focused body, was seen as essential for effectiveness yet potentially limiting. Human health is widely viewed as a necessary structural centre of One Health [33], but the infectious diseases and pandemics focus raises questions about operational attention to non-communicable and non-human aspects [34]. Nonetheless, participants expressed confidence that rotational culture has fostered long-term investment among the five agencies by providing regular opportunity for all agencies to table their agendas, ensuring animal and environmental health concerns remain visible.

These priorities are determined through a consensus-building process participants call “socialisation,” involving multiple discussions and meetings to cultivate “semi-informal relations.” These relations maintain professional boundaries while enabling informal exchanges (e.g., texts, calls, coffee chats, social activities) that facilitate rapid knowledge transfer that has proven useful during public health emergencies requiring multisectoral coordination, affirming that social interaction is foundational to One Health governance [26]. These semi-informal relations support OHO-led bilateral engagements to resolve disagreements, follow up actions, and disseminate information across governance levels. Participants described this dependence on social relations as key to success but acknowledged the challenge of maintaining momentum with recurring personnel rotations.

Multisectoral governance in Singapore’s One Health space is thus predicated on socialising stakeholders. Global literature gaps suggest examining not only institutional health governance structures but also individual actors driving multisectoral collaboration [35]. Our findings extend this by showing that regular inter-agency and bilateral meetings, together with rotational chairing, socialise public servants both formally and informally towards collective care and consensus. Our findings connect to international One Health governance critiques in several important ways. The Quadripartite operational tools for One Health [36] employ predominantly quantitative, indicator-based approaches that may systematically miss the qualitative social processes our ethnography reveals as foundational (trust-building, informal communication, consensus development). Singapore’s experience aligns with critiques that international One Health guidance has limited feasibility when applied to diverse national contexts [37]. A strong, centralised city-state with a compact institutional landscape operates through mechanisms—deliberately cultivated informal networks, rotating leadership, close institutional relationships—that differ substantially from those designed for larger, fragmented, or federally structured contexts. This suggests socialisation processes represent a critical but under-theorised dimension of One Health governance that current assessment tools fail to capture. Future Quadripartite iterations might incorporate qualitative, process-oriented indicators capturing socialisation dynamics alongside structural measures.

Our findings also resonate with broader critiques of ‘global health governance’ models that insufficiently attend to local political and institutional configurations [38]. Singapore’s compact, unfederated system makes the socialisation and rotation we describe feasible, raising questions about suitability for larger or devolved contexts. The deliberate cultivation of informal relationships as a governance strategy may be particularly effective in small, centralised states but may require different adaptations elsewhere. Nonetheless, the insight that formal governance structures can be designed to foster informal relationships essential for multisectoral coordination has potential relevance across diverse contexts.

### Implications for One Health governance

#### Formalising socialisation in health spaces.

Even before CDA establishment, Singapore’s One Health approach prioritised sustainable collaboration over short-term efficiency through “semi-informal” institutionalisation of socialisation, proving invaluable in health crises in which effective response depends on trust and understanding developed in “peacetime.”

While fostering social relations enhances operational effectiveness, it raises sustainability questions. Singapore’s rotational public service culture presents both opportunities and challenges for agenda continuity. Rotation safeguards against over-dependence on individuals, yet some participants observed that collaborative continuity can be shaped by evolving priorities. A key challenge is thus formalising policies, structures, and protocols without eroding social foundations. Bureaucratisation can routinise and dilute ideals that make initiatives effective [39]. Yet inter-agency commitment to embedding socialisation into formal structures reflects determination to retain collaborative ethos.

Maintaining rotational structures while institutionalising bilateral coordination and inter-agency gatherings can nurture future leaders and sustain shared ownership. Strengthening mechanisms that encourage multisectoral socialisation may further embed intrinsic motivation for collaboration and ensure crisis preparedness. These efforts can be codified into enduring policies.

Beyond Singapore, findings affirm that socialisation could be formalised in international One Health guidance. As they are, Quadripartite tools lack capacity to consider context. Assessing One Health requires contextualisation in specific multisectoral governance configurations. Multisectoral socialisation processes help drive complexities across different One Health contexts and must be considered in developing more holistic programmatic literature on One Health.

#### Enhancing multisectoral health governance in Singapore.

Singapore’s One Health approach, emphasising rotating personnel and thus ideas across agencies, facilitates recognition of trade-offs when balancing multiple interests. Social networks serve as conduits for frank discussions that might be more constrained in formal settings, complementing formal processes and reinforcing Singapore’s One Health governance model of consensus-building and shared ownership. While agencies maintain distinct mandates, their workstreams converge. For instance, incorporating vector control within NEA, rather than MOH as in other countries, enables natural convergence between environmental and human disease governance, demonstrating how institutional design complements social networks in enhancing integrated health outcomes.

Beyond One Health, other health-oriented WOG approaches indicate similar multisectoral collaboration. Singapore’s anti-vaping initiative mobilises multiple bodies, including Singapore Police Force (SPF), Central Narcotics Bureau (CNB), Health Sciences Authority (HSA), NParks, NEA, and Immigration Control Authority (ICA) [40], achieving initial successes through joint enforcement and public campaigns [41]. Unlike One Health’s distributed “peacetime” leadership, this initiative is clearly MOH-led, demonstrating how Singapore’s WOG model is configured for crisis response.

One Health integration could benefit several other WOG initiatives. First, to address climate-sensitive diseases offer a natural entry point, as planning decisions regarding biosurveillance in biodiversity-rich areas, environmental management, and urban landscapes directly shape vector-borne disease ecology, human-wildlife interactions, and environmental health exposures [42,43]. Embedding One Health perspectives into sustainability planning would operationalise the environmental health dimension that some participants noted as underemphasised in the current infectious disease-focused approach. Second, as climate change drives more time indoors, reducing indoor pathogen transmission and associated vector risks through built environment redesign and management emerges as an additional One Health priority warranting greater WOG planning and research. Third, food safety initiatives, already involving SFA, MOH/CDA, and NEA, represent another domain in which One Health socialisation could enhance coordinated governance [44,45]. Together, these examples illustrate how embedding One Health within existing WOG initiatives, rather than treating it as standalone agenda, could formalise the approach while building on the social relations this study documented.

Future research could examine how multisectoral socialisation operates in other health-oriented WOG approaches, including systematic rotation and inter-agency collaboration. Comparative analyses could reveal how different socialising structures enhance health outcomes. As CDA matures and new WOG efforts emerge, understanding how internal social networks interact with structural dimensions will be key to strengthening multisectoral governance for health.

### Limitations

Several limitations should be considered. First, to maintain separation between data collection and CDA-affiliated co-authors and avoid untenable power dynamics, a single participant-observer conducted all observations, interviews, coding, and initial analysis. This introduces subjectivities shaped by the researcher’s positionality as a young male governance scholar with limited public health exposure. Mitigation strategies included iterative participant validation (e.g., discussing tentative themes with later participants), sharing anonymised excerpts with CDA co-authors for contextual interpretation while preserving analytical autonomy, and senior author interpretive review of coding decisions. Despite these measures, single-fieldworker ethnography cannot achieve the interpretive consensus of multi-researcher teams. Second, confidentiality concerns may have limited participants’ willingness to share critical views and restricted access to meetings and internal documents, narrowing observations to gatherings deemed appropriate by CDA. Member checking, document review, and interviewing participants about unobserved meetings helped mitigate this. Additionally, maximum variation sampling may have under-sampled critical voices who declined participation, potentially skewing findings toward positive assessments. Third, Singapore’s small size and centralised governance may limit transferability of lessons to larger or more devolved national contexts. The mechanisms identified (rotating leadership, compact institutional landscape, informal networking) may function differently in settings with other governance traditions or political cultures. Lastly, while not a limitation, readers should note that this study documents Singapore’s One Health structure during OHO’s initial development prior to CDA’s official launch, so does not reflect later organisational changes.

## Conclusion

Singapore’s One Health experience demonstrates that effective national health governance depends on actors being well “socialised” with one another. Rotating chairs and personnel institutionalised social networking that supported public health and emergency response successes even before CDA’s formation. The challenge now is in formalising these structures within a maturing One Health bureaucracy and embedding the approach within WOG initiatives without eroding its social core. Singapore’s compact, unfederated system makes such socialisation and rotation feasible, though transferability to larger or devolved contexts remains an open question. Nonetheless, national public health agencies elsewhere can learn from Singapore by identifying and strengthening the social features of their multisectoral collaborations. The Singapore case suggests formal governance structures can be designed to cultivate social processes, an insight with implications for One Health governance and assessment elsewhere. Ultimately, socialisation both enables and is enabled by multisectoral governance. Understanding these reciprocal dynamics is essential to defining optimal health governance models.

## References

[1] World Health Organization. Strengthening multisectoral engagement for health security. World Health Organization. Accessed 2025 January 9. https://www.who.int/activities/strengthening-multisectoral-engagement-for-health-security

[2] World Health Organization, Food and Agriculture Organization of the United Nations, World Organization for Animal Health. Multisectoral Coordination Mechanisms Operational Tool: An Operational Tool of the Tripartite Zoonoses Guide. World Health Organization, Food and Agriculture Organization of the United Nations, World Organisation for Animal Health; 2022.

[3] Government of Singapore. White Paper on Singapore’s Response to COVID-19. 2023. https://www.nas.gov.sg/archivesonline/government_records/record-details/e27f543c-c863-11ed-a758-0050569c7836

[4] Lim V. New Singapore communicable diseases agency to be set up in first half of 2025. Channel News Asia. 2025.

[5] Communicable Diseases Agency. AMR and the National strategic action plan on AMR. 2025. Accessed 2026 July 8. https://www.cda.gov.sg/professionals/antimicrobial-resistance/amr-and-the-national-strategic-action-plan-on-amr/

[6] Lee L. New agency to boost tackling of infectious diseases to be set up by first half of 2025. The Straits Times. 2025.

[7] Food and Agriculture Organization. Contributing to One World, One Health. Accessed 2026 July 8. https://www.fao.org/4/aj137e/aj137e00.htm

[8] Sanga VT, Karimuribo ED, Hoza AS. One Health in practice: Benefits and challenges of multisectoral coordination and collaboration in managing public health risks: A meta-analysis. Int J One Health. 2024;:26–36. doi: 10.14202/ijoh.2024.26-36

[9] Chien Y. How did international agencies perceive the avian influenza problem? The adoption and manufacture of the ‘One World, One Health’ framework. In: Dingwall R, Hoffman LM, Staniland K, editors. Pandemics and emerging infectious diseases: The sociological agenda. New Jersey: Wiley & Sons; 2013. 10.1111/j.1467-9566.2012.01534.x 23095003

[10] Sinha M. Swine flu. J Infect Public Health. 2009;2(4):157–66. doi: 10.1016/j.jiph.2009.08.006 20701877

[11] Sikakulya FK, Mulisya O, Munyambalu DK, Bunduki GK. Ebola in the Eastern democratic Republic of Congo: One health approach to infectious disease control. One Health. 2019;9:100117. doi: 10.1016/j.onehlt.2019.100117 31993475 PMC6976930

[12] Gao S, Zeng Z, Zhai Y, Chen F, Feng X, Xu H, et al. Driving effect of multiplex factors on Mpox in global high-risk region, implication for Mpox based on one health concept. One Health. 2023;17:100597. doi: 10.1016/j.onehlt.2023.100597 38024251 PMC10665165

[13] Kelland K. How climate change increases pandemic risk. Coalition for Epidemic Preparedness Innovations. 2023. Accessed 2025 January 9. https://cepi.net/how-climate-change-increases-pandemic-risk

[14] Abdullah Z. Singapore leads wildlife health efforts as chair of regional network. The Straits Times. 2024. Accessed 2024 November 19. https://www.straitstimes.com/singapore/environment/singapore-leads-wildlife-health-efforts-as-chair-of-regional-network

[15] Goh D. ASEAN One health efforts: Tackling the intersections of climate change and health. S. Rajaratnam School of International Studies. 2024. Accessed 2024 November 19. https://www.rsis.edu.sg/rsis-publication/rsis/asean-one-health-efforts-tackling-the-intersections-of-climate-change-and-health/

[16] Singapore’s one health master plan ONEHealthSG building a resilient tomorrow for people, animals, and the environment. Accessed 2026 July 8. https://isomer-user-content.by.gov.sg/18/906809c5-7e70-410f-a8b5-42003eefe699/Singapore%20One%20Health%20Master%20Plan.pdf

[17] Goudet J-M, Binte Arif F, Owais H, Uddin Ahmed H, Ridde V. Climate change and women’s mental health in two vulnerable communities of Bangladesh: An ethnographic study. PLOS Glob Public Health. 2024;4(6):e0002080. doi: 10.1371/journal.pgph.0002080 38935627 PMC11210765

[18] Dixon J, Dhodho E, Mundoga F, Webb K, Chimberengwa P, Mhlanga T, et al. Multimorbidity and health system priorities in Zimbabwe: A participatory ethnographic study. PLOS Glob Public Health. 2025;5(4):e0003643. doi: 10.1371/journal.pgph.0003643 40293978 PMC12036853

[19] Adeoye‐Olatunde OA, Olenik NL. Research and scholarly methods: Semi‐structured interviews. J Am Coll Clin Pharm. 2021;4(10):1358–67. doi: 10.1002/jac5.1441

[20] Knott K. “Insider/Outsider Perspectives.” Insider/Outsider Perspectives, by John Hinnells. London, Routledge; 2009.

[21] Ow Yong LM, Yi H, Low LL, Thumboo J, Lee CE. A policy ethnography study of a Singapore regional health system on its governance adaptations and associated challenges as a project organisation to implement Healthier Singapore. Public Health Pract (Oxf). 2023;6:100429. doi: 10.1016/j.puhip.2023.100429 37766739 PMC10520508

[22] Errecaborde KM, Macy KW, Pekol A, Perez S, O’Brien MK, Allen I, et al. Factors that enable effective One Health collaborations - A scoping review of the literature. PLoS One. 2019;14(12):e0224660. doi: 10.1371/journal.pone.0224660 31800579 PMC6892547

[23] Abbas SS, Shorten T, Rushton J. Meanings and mechanisms of One Health partnerships: Insights from a critical review of literature on cross-government collaborations. Health Policy Plan. 2022;37(3):385–99. doi: 10.1093/heapol/czab134 34791224 PMC8896336

[24] Comaroff J, Comaroff J. Ethnography and the Historical Imagination. New York: Routledge; 2019.

[25] Robinson RS. Purposive Sampling. Encyclopedia of quality of life and well-being research.Dordrecht: Springer; 2014. https://link.springer.com/rwe/10.1007/978-94-007-0753-5_2337

[26] Laerd D. Purposive sampling. Accessed 2023 October 1. https://dissertation.laerd.com/purposive-sampling.php#maximum-variation-sampling

[27] Braun V, Clarke V. Thematic analysis: A practical guide. London: Sage; 2021.

[28] Khurshid F, Veen M, Thompson J. Navigating thematic analysis: Practical strategies grounded in abductive reasoning. Teach Learn Med. 2025;1–10. doi: 10.1080/10401334.2025.2475098 40172437

[29] Thompson J. A guide to abductive thematic analysis. The Qualitative Report. 2022;27(5):1410–21.

[30] Colebatch HK. Making sense of governance. Policy and Society. 2014;33(4):307–16. doi: 10.1016/j.polsoc.2014.10.001

[31] Ye R. Schooling for government: institutionalised sponsored mobility and trajectories of public service scholarship recipients in Singapore (1979–2018). J Edu Work. 2021;34(4):518–32. doi: 10.1080/13639080.2021.1943335

[32] Poocharoen O, Lee C. Talent management in the public sector: A comparative study of Singapore, Malaysia, and Thailand. Public Management Review. 2013;15(8):1185–207. doi: 10.1080/14719037.2013.816525

[33] Su Z, McDonnell D, Cheshmehzangi A, Bentley BL, Šegalo S, da Veiga CP, et al. Where should “Humans” be in “One Health”? Lessons from COVID-19 for One Health. Global Health. 2024;20(1):24. doi: 10.1186/s12992-024-01026-y 38528528 PMC10964596

[34] Wallace RG, Bergmann L, Kock R, Gilbert M, Hogerwerf L, Wallace R, et al. The dawn of Structural One Health: A new science tracking disease emergence along circuits of capital. Soc Sci Med. 2015;129:68–77. doi: 10.1016/j.socscimed.2014.09.047 25311784

[35] Shankardass K, Solar O, Murphy K, Greaves L, O’Campo P. A scoping review of intersectoral action for health equity involving governments. Int J Public Health. 2012;57(1):25–33. doi: 10.1007/s00038-011-0302-4 21931976

[36] Multisectoral coordination mechanism operational tool. Accessed 2026 July 8. https://www.who.int/initiatives/tripartite-zoonosis-guide/multisectoral-coordination-mechanism-operational-tool

[37] Traore T, Shanks S, Haider N, Ahmed K, Jain V, Rüegg SR, et al. How prepared is the world? Identifying weaknesses in existing assessment frameworks for global health security through a One Health approach. Lancet. 2023;401(10377):673–87. doi: 10.1016/S0140-6736(22)01589-6 36682374

[38] Sen K, Qadeer I, Missoni E. Understanding the context of global health policies. World Rev Political Economy. 2022;13(3).

[39] James N, Field D. The routinization of hospice: charisma and bureaucratization. Soc Sci Med. 1992;34(12):1363–75. doi: 10.1016/0277-9536(92)90145-g 1529374

[40] Whole-of-government efforts to tackle vaping. Accessed 2025 September 30. https://www.moh.gov.sg/newsroom/whole-of-government-efforts-to-tackle-vaping

[41] Dhanya V, Vimalan D, Yiing LS. Health Promotion Board uses toxic toys to warn against vaping. 2025. Accessed 2025 September 30. https://www.marketing-interactive.com/hpb-uses-toxic-toys-to-warn-against-vaping-risks

[42] SG Green Plan. Our global commitment. 2020. Accessed 2026 July 8. https://www.greenplan.gov.sg/globalcommitment/

[43] Ministry of Sustainability and the Environment. Climate game changer. 2025. Accessed 2026 July 8. https://www.mse.gov.sg/policies/climate-change/climate-game-changer/

[44] Singapore Food Agency. Securing Singapore’s food safety through our collective commitment. Food For Thought. 2024. Accessed 2026 July 8. https://www.sfa.gov.sg/food-for-thought/article/detail/securing-singapore

[45] National Environment Agency. Food Waste Management Strategies. Accessed 2026 July 8. https://www.nea.gov.sg/our-services/waste-management/3r-programmes-and-resources/food-waste-management/food-waste-management-strategies

